# Bereaved parents involvement in maternity hospital perinatal death review processes: ‘Nobody even thought to ask us anything’

**DOI:** 10.1111/hex.13645

**Published:** 2022-11-06

**Authors:** Änne Helps, Keelin O'Donoghue, Orla O'Connell, Sara Leitao

**Affiliations:** ^1^ Department of Obstetrics and Gynaecology, Pregnancy Loss Research Group University College Cork Cork Ireland; ^2^ Department of Obstetrics and Gynaecology, INFANT Research Centre University College Cork Cork Ireland; ^3^ Department of Obstetrics and Gynaecology, National Perinatal Epidemiology Centre (NPEC) University College Cork Cork Ireland

**Keywords:** bereaved parents, maternity hospital death reviews, parents involvement, perinatal death, stillbirth

## Abstract

**Introduction:**

The death of a baby is devastating for parents, families and staff involved. Involving bereaved parents in their baby's care and in the maternity hospital perinatal death review can help parents manage their bereavement and plan for the future. In Ireland, bereaved parents generally have not been involved in this review process. The aim of our study was to assess parents' perception of how they may be appropriately involved in the maternity hospital perinatal death review in ways that benefit them and the review process itself.

**Methods:**

Bereaved parents (*n* = 20) in Ireland were invited to take part in semistructured interviews. Thematic analysis was carried out on the interview transcripts.

**Results:**

Four main themes were identified based on the participants' views and opinions on how they experienced the review process and how they feel this process may be improved. The themes reflect the journey of the parents through the different stages of the review process: Throughout process; On leaving the hospital; Interaction with the hospital ‘waiting in limbo’; Review itself. Identified subthemes highlighted essential aspects of this process and care provided to parents. For the parents, open, honest communication with staff, as well as having a key hospital contact was essential. Parents wished to provide feedback on their experience and wanted to be included in the review of their baby's death, in a way that was sensitive to their needs and the hospital's schedule.

**Conclusion:**

A respectful, flexible system that allows bereaved parents' involvement in their baby's perinatal death review and is tailored to their needs is essential. A collaborative process between staff and parents can highlight clinical areas in need of change, enhance lessons learned, improve bereavement services and may prevent future perinatal deaths.

**Public Contribution:**

Bereaved parents were interviewed for this study.

## 1 INTRODUCTION

The death of a baby during pregnancy or shortly after birth is devastating for parents and families and can deeply affect the healthcare staff involved. Unfortunately, some deaths are inevitable (e.g., due to a fatal foetal abnormality) but others may be preventable if significant risk factors are recognized antenatally (i.e., during pregnancy) or intrapartum (i.e., during labour). After a perinatal loss (stillbirth or death within 4 weeks after birth, i.e., neonatal death), parents commonly experience negative psychological symptoms which can persist into subsequent pregnancies.^1^ Acknowledging the importance of the deceased baby as an individual and involving the bereaved parents in all aspects of the baby's care (such as washing, dressing and examinations if appropriate) can help the parents process their bereavement and plan for the future.^2^, ^3^, ^4^

The purpose of local child death reviews, like the ones carried out after a perinatal death (stillbirth or neonatal death) in maternity hospitals, is to gather all the information on events relevant to the death, identify contributory factors and cause of death, and to recommend changes to prevent future deaths in maternity hospitals by identifying and addressing modifiable risk factors.^5^ The bereaved families should be treated with compassion and be offered the opportunity to be part of the review process.^5^, ^6^ A study examining parental involvement in perinatal mortality review processes in six high‐income countries found procedures were not established, and only 1 in 20 of the 1104 participating healthcare professionals described a detailed approach to parental engagement in reviews.^7^

In the United Kingdom, the PARENTS 1 and PARENTS 2 studies examined how bereaved parents want to be involved in the local perinatal review process and how this can be achieved.^2^, ^8^, ^9^, ^10^ The PARENTS 1 study showed that bereaved parents want to be part of the perinatal review process in a way that is ‘open and transparent, and emphasised the need for an inclusive and positive approach to both medical and emotional aspects of care’.^2^, ^10^ Many benefits of involving parents in reviews were identified, such as the parents providing additional, relevant information to the process; helping the parents to understand events around their baby's death, and encouraging the hospital to learn lessons and change practices accordingly.^8^, ^10^ Barriers to parental involvement in reviews mentioned in previous studies included the cost involved and fear of litigation,^7^ a language barrier between some bereaved parents and professionals^11^ and variations in bereavement care service provision across maternity units.^10^

The Perinatal Mortality Review Tool (UK‐PMRT) was launched in 2018 to standardize perinatal mortality reviews across the United Kingdom and to ensure bereaved parents are always involved in the review of their baby's death.^12^, ^13^ Specific material is readily available to facilitate parental engagement in reviews using the PMRT.^14^

In Ireland, bereaved parents generally have not been invited to be involved in the perinatal death review, as the current process in place does not facilitate their involvement.^15^ Instead, the final results and findings are usually discussed at the parents' follow‐up visit with their consultant.^16^ Of note, the National Incident Management Framework published in 2018 by the Irish health service stated that families must be informed if a review is going to be carried out and should be given the opportunity to give their perspective of events.^17^ However, in Ireland, there is no specific guidance on involving bereaved parents in review processes specifically.

A study from 2019 showed that just over half (58%) of Irish maternity units regularly informed bereaved parents of the local perinatal death review taking place.^18^ Furthermore, only 17% of Irish maternity units stated that the final review report was provided to the bereaved parents.^18^ A study on 10 inquiry reports relating to perinatal deaths and pregnancy loss services in Irish maternity services stated that only 40% of the inquiries involved all of the affected families.^19^

This study aimed to learn from and with bereaved parents, how they may be appropriately involved in the local maternity hospital perinatal death review process in Ireland in a way that is beneficial to both them and the review process itself.

## 2 METHODS

### 2.1 Recruitment

Bereaved parents from all regions in the Republic of Ireland were invited to participate in the study. Purposeful sampling was implemented to recruit bereaved parents in collaboration with Clinical Midwife Specialists in Bereavement Care and parent representatives working within Voluntary Organizations supporting bereaved parents. These acted as a liaison to bereaved parents who had experienced a perinatal death (stillbirth or neonatal death), informing these potential participants about the study over the phone or through emails. Inclusion criteria included parents who were over 18 years of age, spoke fluent English, were at least 6 months postperinatal bereavement (stillbirth or neonatal death) and had no more than 6 years since completion of their child's death review. Previous research with bereaved parents showed that 6 months after their bereavement was an acceptable timeframe for parents to be approached about research participation.^20^

Once a parent gave consent to be contacted, they were contacted by email or phone by one of the researchers with a personal invitation to participate in a semistructured interview. Each participating parent was invited to extend the invitation to participate to their partner. Recruitment occurred between October 2020 and March 2021.

### 2.2 Setting

There are 19 maternity units in the Republic of Ireland, which are funded through the Department of Health.^21^ The maternity units vary significantly in size and activity; with between 1000 and 9000 babies being born per annum.^21^ The majority of births (>90%) in Ireland occur in the hospital setting, under consultant‐led care.^21^ To maintain anonymity the parents were not asked which hospital their baby was born and/or died in. To ensure representation from all regions in Ireland, the parents were asked which province they lived in.

### 2.3 Interviews

Semistructured interviews were carried out at a time convenient for the parent(s). A topic guide was used with open exploratory questions to encourage a conversational flow and allow participants to express their experiences, views and opinions on how, when and where parents would like to be and can be involved in the local review process in their and their baby's care.

Before recruitment began, a pilot interview was carried out with a parent representative from the local pregnancy loss research group to check the topic guide for clarity. All terminology was confirmed to be sensitive to the parents' bereavement during the pilot interview. This interview was not recorded and was not included in the analysis.

All parents were offered to have interviews carried out individually or with their partner present (sometimes for support) according to their preferences. The interviews took place between November 2020 and March 2021. Due to the COVID‐19 pandemic and national public health guidance, all interviews were carried out remotely using a virtual meeting platform. Specific interview protocols were established to ensure security. The interviews were semistructured, lasted between 27 and 107 min (median, 58 min), were audio‐recorded using a Dictaphone and transcribed verbatim.

### 2.4 Analysis

Data collection and analysis were conducted simultaneously. A qualitative research design was used to identify and report patterns in the data and to describe them in rich and meaningful detail.^22^ The data analysis methodology was based on the principles of reflexive thematic analysis as described by Braun and Clarke and followed their six‐phase process.^22^, ^23^ First, all transcripts were anonymized, read and reread by the first author to become familiarized with the data and identify initial codes. Second, open, systematic coding facilitated the researcher to identify codes (and quotes) related to the research objective. Six of the interviews were read and coded independently by two of the other authors (three each). The three researchers with the aid of thematic maps discussed, reviewed and grouped the initial codes to reach a consensus and actively generate the main themes and related subthemes. The transcripts were then re‐examined by the first author to ensure all relevant and poignant data extracts were included and fitted within the generated themes and subthemes. Two of the authors discussed, further developed and refined the themes and subthemes to generate clear definitions and names for each, as well as clarify the overall flow of the analysis of bereaved parents' involvement in hospital reviews. Finally, these themes and subthemes were reviewed and agreed on by all authors. The four final themes are united by a central concept (i.e., the bereaved parents' journey through their review process) and the subthemes share patterns of meaning within each theme.^23^ An audit trail of the phases of continuous analysis was kept.

## 3 RESULTS

### 3.1 Participants

**Figure 1:**
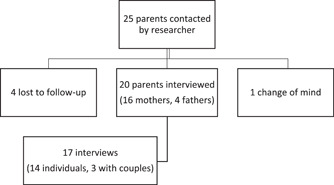
Recruitment to interview process

Twenty‐five parents were contacted by the researcher, 20 of whom participated in 17 semistructured interviews (Figure 1). In total, 16 mothers and 4 fathers were interviewed. Ten of their babies were stillborn and six died in the neonatal period. It was at least 6 months since their bereavement for all parents (median 3.5 years). There was representation from three of the four provinces in Ireland, as well as from regional and tertiary Irish maternity units.

Results are reported on the lived experiences of the parents and their views on how meaningful engagement by parents in review processes may be achieved, as well as the reasons why this is important.

**Figure 2:**
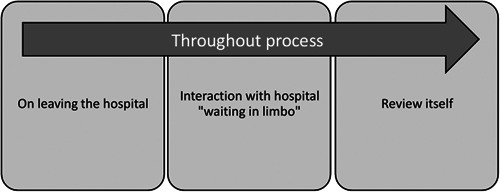
Outline of themes in the study

Four overarching themes were identified from the data (Figure 2). Three of the themes represented different (though at times overlapping) stages of the bereaved parents' journey through the hospital review process, and the fourth theme ‘throughout the process’ contains subthemes that were important and relevant throughout the whole journey (Figure 2).

The 13 subthemes stemming from the four themes are presented in Table 1. Direct quotes from the interviews (indicated by ‘*Interview*’ and the interview number) are used to highlight each theme. Short quotes are present within the main text (and subheadings); further quotes are presented in Tables 2, 3, 4, 5.

**Table 1:** Themes and subthemes identified from the interviews

Themes	Subthemes
Throughout process ‘an informed approach is a fair approach’	Impact of grief on parents
	A just, compassionate culture with honesty
	Importance of communicating with parents with regular updates
	Support for parents
On leaving the hospital ‘you're just given so much information inside the hospital’	Information given to parents (verbal and written)
	Having a point of contact/key contact
Interaction with hospital ‘waiting in limbo’	The follow‐up meeting for parents
	Parents providing feedback to the hospital
Review itself ‘a way to get answers’	Aims of reviews
	Parents' contribution to reviews
	Delivering information to parents
	Inconsistencies for parents with reviews
	Outcomes of the reviews for the hospital and the parents

**Table 2:** Theme ‘Throughout process’

Subtheme	Quotes
Impact of grief on parents	1. …, when you're leaving the hospital, you're in a kind of a (pause) eh haze. …you're not able to take things in and you have questions afterwards, you know, after, you know, maybe a couple of weeks after that you you, you're kind of you have further questions that you are like, oh I should have asked that … I can't remember, you're told things as well, but you can't remember them because you're, … you're totally (pause) consumed with grief. (*Interview 8*)
	2. You're going up for facts and you come away with em (pause) … kind of worry and you know you're sent down a different, a different road. Thinking I wasn't that before but now I am, you know, and that's grief and trauma too, eh grieving process, it's a very up and down road. So you need the medical side to be consistent. (*Interview 2*)
A just, compassionate culture with honesty	3. But even now every day if I have time, if I go to the hospital or if I am passing the hospital I always have a warm feeling about it because of the way the staff were up there. And even down to the explaining the process of what the pathologist does and the coroner was all done very naturally and there was warmth in it, there was no talking about clinical things and all that kind of stuff. (*Interview 14*)
	4. …she was fantastic I have to say, I got very lucky with the lead on our review, just with her compassionate empathic approach. She was fair to all sides, … she made sure that all sides were appropriately met with fairness and justice. (*Interview 13*)
	5. We had a list of questions we wanted to ask, they weren't hard, they were basic questions about my care, about the systems that failed us. We just wanted the simple answers to those. And we firmly believe if staff could engage, if there was the culture in place for this to go together … where it is protected, where we can all sit in a room, for once we could get the answers that we were looking for. (*Interview 16*)
	6. And you know, obviously if there's, if there was an issue, if they were able to tell us the truth and, and you know from the very beginning obviously that would have been better and then we could have gone away I suppose, and, and try and absorb that. (*Interview 11*)
	7. I suppose my big issue with the whole situation was the lack of information, literally we had to keep asking and asking. It was like trying to get blood from a stone. They wanted to be open and transparent but they wanted to be open and transparent if it suited them. I found the amount of information that was hidden, that was underhanded was an absolute joke. (*Interview 17*)
Importance of communicating with parents	8. So I was always kept informed. It is so important. An informed approach is a fair approach and that needs to be taken with bereaved parents … Like this is the death of a child, it is an ongoing process and all you are doing is sitting at home waiting for some sort of information. And the information that is received and the information that is given to bereaved parents, it is not enough. It is not okay to just send a one sentence email to say, yes they are still working on it. (*Interview 13*)
	9. So I suppose we wanted to know why and how, some people don't want to know that and that is entirely up to them. … all parents should be given the information whether they want to act on that information I suppose is entirely up to them, but they should definitely be given the option. (*Interview 17*)
	10. She rang a few times and then she was texting and stuff just to see how we were getting on. And I suppose, only because I kind of a good rapport built up with (name). I felt I could text her and ask her, you know, questions … And I mean, she would be great, like you'd never be, you would never be waiting long for her or anything like that. (*Interview 7*)
	11. …for me it just seemed like (pause) there hasn't been and there's still not any kind of appetite to hear my views. And like, I kind of feel like even though I've only sent a few emails, but I still kind of feel like I'm almost pestering to try to get things reviewed properly, and like our baby died! You know, I, I don't think I should be the one to have to keep following up, to try to make things be done properly … Like you know, obviously, if I wasn't trying, there'd be zero interaction. And even when I am really really trying, it's slow and it doesn't feel like that people really want to engage or listen really. (*Interview 5*)
Support for parents	12. I think the only time that I really felt I could have used more support was when I was discharged. Because you're going from a circumstance whereby you have midwives around you the whole time looking after you. You know, and you're getting fed at certain times. … you have this really good support bubble, you're wrapped in cotton wool, and then suddenly you're sent out to the real world and you have to stand on your own two feet and you're grieving. (*Interview 4*)
	13. I think the hospital should make contact with the parents and be like, you know, look, the support is here if you need it, like, you can contact us when you're ready like … D'you know, eh would you like to talk about this? Would you, like, give them time, but also give them the option that there is always someone there to talk to. Like when they're ready. (*Interview 6*)
	14. And like, she wasn't pushy. She rang all right. … But after that, then she'd text. So, like, if you didn't feel like talking, that was fine. No, she wasn't em, she wasn't pushy at all, at all. You know, you appreciated the phone call the first week because everything was so new and she was, but … Some people might be more private. I don't know. But I definitely liked (name of bereavement midwife) checking in. (*Interview 7*)
	15. So we didn't really know what to expect or what to do. But in hindsight, only for our bereavement midwife at the time. She guided us through all of that. Our consultant didn't ask to meet with us. (*Interview 3*)
	16. But that support *(from patient advocate)* continued all the way along throughout the years. She would always pop in every now and again and say how are things. Or if there was something I needed to ask about that I was very unsure about myself it was only a matter of picking up the phone. (*Interview 13*)

**Table 3:** Theme ‘On leaving the hospital’

Subtheme	Quotes
Information given to parents (verbal and written)	1. …because sometimes you're just given so much information inside in the hospital. I think maybe even a follow up, a call … two or three weeks later, just to kind of nearly check in and see, ‘do you want to have the follow up, do you want to have … these are the numbers available, do you want to come and have a meeting?’ Here, you know, I think you're just given so much information in the hospital, sometimes you kind half forget, you know, there's so much coming at you. (*Interview 8*)
	2. I think it should be arranged before you leave the hospital, just to say that this is coming down the line. The results will come back. They may show something or they may not show something. Em would you, I mean, would you like to meet with us? (*Interview 3*)
	3. There must be some two or three steps that could be, could be time‐lined and person specific and explain, given to the parents on paper to go this is what the hospital will come to you with. (*Interview 2*)
	4. I think there needs to be a booklet developed and that for information, that they are given to families, the coroner process, inquest, investigation, the parents' rights, advocacy support services. But it needs to explain the whole lot, even the terms of reference, as simple as that, I didn't understand enough back then and I know from listening to others that they didn't either. (*Interview 15*)
	5. So I think, you know, have their doctor sit down with them the day they're being discharged and say, look, these are the, you know, supports available to you. And here are the numbers and someone will be in contact with you from these supports, you know, to know if you want to talk or when you're ready, you can reach out and talk yourself. (*Interview 6*)
	6. I would have appreciated that before I left the hospital and I would have appreciated if they had already decided they were going to do a review, a hand out about what this process is. And that as parents you can contact this person at the patient advocacy service and they are there only to support you … And had we had that we would have known what to expect. (*Interview 16*)
Point of contact/key contact	7. And just to, you know, be introduced to each other and say this is your, you know, if you feel that you want to have this contact in the hospital, then, yeah, I think that's important. That's very important I think. (*Interview 11*)
	8. So I suppose like, she was kind of, like if there was anything, I'd probably go to her before somebody else, because (pause) I don't know, I suppose like, you don't really know otherwise who to contact. Em so, yeah, it is important to have a key, you know, probably a single point of contact who maybe could follow up on some things. (*Interview 5*)
	9. Em to have someone, you know, just to be able to, like a key worker or something like that, just so that you're able to talk to someone about the situation and be like, ‘OK, what's what's happening with this’ or ‘how is this gonna go…’ (*Interview 6*)
	10. …I suppose, like there are the bereavement midwifes up there. Em so it's, it probably just needs to be a little clearer to the parents though, like em, who is my contact person if I want to follow up on anything that's happened? (*Interview 2*)

**Table 4:** Theme ‘Interaction with hospital “waiting in limbo”’

Subtheme	Quotes
The follow‐up meeting for parents	1. I would have liked to have had a meeting sooner after we lost (name of daughter), because as I said, like from the minute she died, I was in overdrive. … Em And I would have loved to have sat down with (name of consultant obstetrician) sooner and just been able to just converse with her about it. (*Interview 4*)
	2. And it was now time to start slowly picking ourselves up a little bit. And moving forward with her. … the time was right for us. And we were ready to meet him … So I think a time frame of maybe 6 to 12 weeks, or definitely 12 weeks post, was a good time for us. (*Interview 3*)
	3. Definitely, em I'm not sure if 6 weeks is long enough. I think em parents need longer, longer to try and process everything … And this just on top of having all the normal hormones that you'd have after having a baby. Em I think maybe a longer space of time before, before that discussion is maybe had. Even if it was another month added, you know. (*Interview 11*)
	4. Em I felt like maybe they should have done it in another ward or another floor … it just, it was just horrible, like I was shaking, my whole body was shaking … Like, it just brought back so many memories. Maybe if they are having their meetings and stuff. Maybe they should be on a different floor or over in (name of hospital) in another room like, you know, rather than going back into the maternity hospital, where you know your baby, you had your baby there…. (*Interview 9*)
	5. Well, I know she said that she felt initially coming back into (name of maternity hospital) we'll be upset you know, that she could set up something outside of the hospital … I'm so glad she set it up in the hospital because the day we went in, we met a nurse that looked after me, we met another midwife. And it really grounded us again, to say that (name of daughter) was real and that it did happen and we did deliver her here in the hospital. So initially put my foot inside the door, I did get upset. … But after that it was quite a safe, comfortable place and it was a safe place to go. (*Interview 3*)
	6. So when I met with him, he checked with me. … So just really lovely. You know, he had offered his time. He was very respectful about me as the grieving parent. So, so he, he basically checked in saying, ‘what way would you like to do this? Would you like me to, would you like to ask me questions? Would you like me to run through what happened?’ He's like, ‘just tell me what you need’. (*Interview 2*)
	7. If you are feeling that in the moment you can, you have someone there to support you, like your partner can feel like that as well, so you kind of need someone, either a family member or someone neutral like a patient advocate or someone there with you, I would think is a good idea. We never went to a meeting in the hospital on our own, ever. (*Interview 14*)
Providing feedback to hospital	8. But like you'd love them to know exactly how good their staff were, you know. Em yeah no, I suppose like there isn't really the opportunity to, to say any of that. Like when you go for, when you're getting the results even like it's, it's, it's very medical you know, you're only talking about results, future pregnancies. Like the last thing you'd be thinking about is being like, oh, ‘by the way, I had a great experience, thank you’. (*Interview 7*)
	9. I see that to give feedback would be great. If anything I can do to help other parents going through this and to prevent, I suppose, certain things that happened for us, not that we were met with much negativity to be honest. … Em yes, we would have loved that. Em and I suppose not just a letter but to be met, (pause) and to, to give our side of things or what we were unhappy with, or happy with … Most definitely, I think for moving forward and closure and for grieving, it would be very important on both sides, to get both sides of the story. (*Interview 3*)
	10. They were listening to us. Like we spoke to them for two hours … Em but kind of highlighting all of the things that we felt, you know (pause) possibly could have made a difference. And we haven't really got response on some things and, you know, kind of highlighting the things, the areas where we thought there might have been kind of gaps, em and not just for us. Like just in general, you know, like we were kinda saying, ‘look we're not experts, we're not trying to tell anybody what to do, but, this is kind of our experience’. (*Interview 5*)
	11. Like maybe a couple of months down the line, not really straight after, because, you know, like especially if a mother is angry, they're going to say, ‘I hate this, I hate that’, you know what I'm saying? So, like, give it a couple of months and then, like, you know, phone them up and ask them. Or if they're meeting up with someone on the bereavement team, you know, get the person on the bereavement team to be like, ‘look were you happy with the level of care you received and your baby received at this time. And if, if not, how could we change that in the future?’…. (*Interview 6*)
	12. Again I would think around the 12 weeks, you know, let you process everything again, let the hormones settle down … So I think definitely let all that settle after the couple of weeks and then you would be able to speak up. And you would have time to process what has happened as well and speaking with your partner and stuff, he would have picked up things that you mightn't have picked up on. (*Interview 10*)
	13. Maybe even a questionnaire or something because maybe people would be more confident to say things on a questionnaire or an email or something than they would face to face. People might shy away. I would have no problem speaking up for myself but not everybody would … There could be a comments section at the end then if people did want to put in their own little, because obviously everyone's journey was a bit different and their experience. So whether they wanted to express their anger. (*Interview 10*)
	14. I don't know how, like I suppose the, if there was a kind of a follow up meeting that it would, that would be part of that follow‐up meeting, you know. Em if the bereavement midwife, whoever it is or whoever meets to go through, to meet to see if you are, you know, how you are doing, to talk to as part of that process, get feedback there, d'you know em. It, through that, that way I think would probably be a good idea. (*Interview 8*)
	15. Because, like, OK, not everybody might be used to doing emails or, you know, sometimes talking on the phone isn't, well some people might find it easier to do it on the phone, other people might do it face to face. So, yeah, I think an option. I think there should be whatever option a parent wants, really. Like I don't think it should be restricted to just, you know, contact this number between these times or something like that. I think em yeah, just an option of different ways to contact somebody would be good. (*Interview 5*)

**Table 5:** Theme ‘Review itself “a way to get answers”’

Subtheme	Quotes
Aims of reviews	1. As a parent I suppose you want to be your child's voice and I think the review process for a parent, as I said, is a way to get answers to something that (a) they don't understand because it is all medical, and (b) it is giving them closure. (*Interview 14*)
	2. And at every single meeting we kept saying that this is about a systems failure, systemic systems failure where improvements could be made, where this was not to happen to another family. And that is what was most important. (*Interview 16*)
	3. I suppose how it came across to us, was almost they were covering themselves. Unfortunately. But obviously, I know it's to assess what has taken place…. (*Interview 2*)
Parents' contribution to reviews	4. Well to get our side of the story, first of all, because it was a very one sided review, they only got what the doctors and nurses involved. So there was no statement from us at all. … and, you know, for the doctors and nurses involved, em you know, obviously they're not going to try and and, em you know, say anything bad about themselves. There are, so they didn't get the full picture … I think mainly just to write down from our side of things what exactly happened and just explain exactly from our side of things. (*Interview 11*)
	5. I know it's not going to be, in most cases medical information, but it's relevant … And like I was trying to (pause) kind of complement my notes as opposed to contradict them, like I was trying to give more information for it to be reviewed properly … And it's like, like I know most parents probably aren't doctors, but I mean, it's not just the kind of soft, emotional side of it that we can give, like a lot of the time it's actual proper information as well…. (*Interview 5*)
	6. But, you know, just ask parents, like, would you like to provide any further information, you know. I don't know … giving a form would work or, you know, give people just an opportunity, at least ask them at some point, you know, would you like to give any more information? Do you have any other information that you'd like to have included in anything? (*Interview 5*)
	7. …I find it easier for writing down information, personally. I know everybody wouldn't be the same, em (pause) maybe to write it down and then, you know, when they have seen that written statement or, and then maybe set up a meeting with everybody involved, then. (*Interview 11*)
	8. And then it's up to the parents, obviously, whether they decide to be part of it or not. And again, that it needs to be very clear and honest, because the parents need to know what they're getting into obviously. …So as long as the parents, whatever the parents are told they're going to be involved in, is what they're involved in. They're very clear what they're agreeing to. (*Interview 2*)
	9. It was months really trying to get all it sorted, so I think as a rule the system should be changed … because it is a traumatic time of your life and not many people want to go over this. Because every time we went over it, it was heart‐breaking and it was very hard in the days afterwards we found because it is constantly on your mind. You give your account and then a few days later you weren't right really, we both found it, it was very hard. (*Interview 17*)
Delivering information to parents	10. There was no warning you will get it next week, nothing, there you go, in your in‐box. So to say the least that was extremely hurtful and extremely disrespectful to a family. (*Interview 16*)
	11. I think to get the results in the post, if it was me on my own, hormones raging, no baby here at home and to open a letter with the results. I think my (pause) the ground would just opened beneath me again. And it would have just added to my extra grief. The fact that (name of bereavement midwife) phoned me and said, ‘would you like to come and collect them and bring somebody with you?’ And to meet her personally and just the touch of her hand and just be able to get the results into my hand, helped. It really did help. (*Interview 3*)
	12. Em so for us, initially getting our post‐mortem result, we still came home with a lot of weight on our shoulders, whereas the second time round meeting with (name of consultant obstetrician), it was totally different. We came home with our bag was empty. We didn't feel that burden. So I think delivery of the information and how we're met as parents, grieving parents and that our child is acknowledged throughout the meeting. (*Interview 3*)
	13. They gave it to us in the meeting. We had a patient advocate with us and that is when she kinda said, ‘one second now we need the room to go through this’. If we didn't have that I don't know would we have got anywhere, would we have got half of what we needed to get out of it. (*Interview 17*)
	14. But I definitely do think that if parents provide feedback, then it should be, you know, reviewed properly and noted and maybe give parents an opportunity to em review the report. And for that not to be a kind of a final report, maybe provide something to feed back into it and then finalize the report or something like that. (*Interview 5*)
	15. …we had to constantly write, after three or four months, guys what stage is this at now? When it is supposed to take the 120 days, to take the length of time it took is just insane for the actual report that we got in the end. (*Interview 16*)
	16. And I will never know the answer to that. That was one thing that really upset me, I really thought that by getting a review I would have all the answers … That was the hardest part of it all I think because when you get a review as a parent you expect all your questions to be answered. Because they tell you that is what it is going to be. (*Interview 14*)
	17. So like these people, they must think that you're never going to look for freedom of information. You're never going to get all your files. You're never going to read all these emails. Like, yes, there's an awful lot of emails but, oh, my baby's dead. So I had time to read them. And as difficult as that was, I've read them. (*Interview 12*)
Inconsistencies for parents with reviews	18. You don't know what's what like, and I was asking his opinion. He basically told me ‘oh reviews are not really worthwhile’. And I was like, really? Because I have a lot of unanswered questions here like, and you're here telling me this. (*Interview 12*)
	19. Em it did say patient concerns were noted, but there was no (pause) eh no detail as to what my concern were. Em or how they had been noted or how they had been reviewed at all … So, you know, I'd kind of really tried (pause) to give as much information as we could. And the only reason I was trying is because I wanted … a full review of (name of son) dying. Like, it's nothing to do with anything, it was literally just, I had information to give. I tried my best to give that information. And then even when I was kind of, you know, going out of my way to follow up and provide all the information that I could. That then was just completely ignored, you know, and it's just, it's just really annoying … I just kind of felt like we were just completely taken out of it, even though it was us and our baby who died. … it's like nobody even thought to ask us anything. (*Interview 5*)
Outcomes of the review for the hospital and the parents	20. …it wasn't like that for us in terms of, we know when staff get up that morning it wasn't their intention to not look after you, it was never their intention. They are human, they make mistakes but the biggest thing from mistakes is learning from them. (*Interview 14*)
	21. But then you are looking at where these recommendations go, who is in charge of overseeing that these recommendations will be actually carried through? So it makes an absolute … What is the point in doing these investigations when they go into a drawer basically? (*Interview 16*)
	22. And that was really amazing that, like, I felt like that was because of our little boy, that he inspired change and that would have been a really lovely thing to hear. I can understand how you have to be careful how you say things to parents, because there are people who sue for anything or anything. But I just thought that was very lovely to hear. (*Interview 2*)
	23. And you don't want the same thing to happen to somebody else, so I think if a parent can, you know, knows themselves that they can give a bit more information and know or think at least that maybe that information might help somebody else. Or help, you know, so that another situation of the same or a similar kind of occurrence … that they might be able to stop that happening. So I think a lot of it is down to trying to (pause), do something for their baby because, like our babies have died. There's nothing really that we can do for them now. But I think for most parents, you know, you probably want to do something kind of for them so that it's not gonna happen to somebody else. (*Interview 5*)

### 3.2 Throughout process ‘an informed approach is a fair approach’

#### 3.2.1 Impact of grief on parents

Grief and its impact was a core experience that was mentioned by the bereaved parents. The parents expressed how grief has an enormous impact on them (‘grief is a killer’, *Interview 1*) and how it affected them both physically (‘we weren't eating properly, we weren't sleeping’, *Interview 11*) and mentally (‘we were very raw’, *Interview 3*). Some described their state of mind like a ‘haze’ or ‘being in a dream’ and ‘in total shock’, ‘totally consumed with grief’. Grief further impacted the way the parents were able to absorb the information given to them and communicate with professionals (Table 2, Quotes 1 and 2). The participants felt that this needs to be taken into consideration when interacting and communicating with bereaved parents throughout the review process.

#### 3.2.2 A just, compassionate culture with honesty

A compassionate culture within the hospitals and the supportive manner of professionals helped parents cope with their bereavement. The hospital culture affected how the parents were able to manage their grief and process the events around the birth and death of their baby, as well as navigate the investigations and reviews that followed (Table 2, Quotes 3 and 4). Parents expressed their gratitude when they met kind, supportive staff: ‘they were just amazing and I can't put it in words how good they were to us’ (*Interview 3*). Whereas those parents that encountered a cold, uncaring environment described the detrimental consequences this had on them: ‘I can tell you the disappointment through the whole thing was their care and like, those words. I regularly get night terrors. I relive that whole experience’ (*Interview 12*).

Being able to ask questions and get answers (‘why did it happen’, *Interview 4*) was essential to the parents (Table 2, Quote 5). The majority of parents expressed how important honesty and openness from professionals were to them from the beginning (‘…if there was an issue, if they were able to tell us the truth and, and you know from the very beginning obviously that would have been better…’, Table 2, Quote 6) and throughout their bereavement journey. Those that were confronted with dishonesty or were ‘kind of pushed aside’ said it made them feel ‘very confused’, ‘suspicious’ and ‘paranoid’. Two parents explained that they felt that openness was on the professionals' terms only (‘…literally we had to keep asking and asking. It was like trying to get blood from a stone…’, Table 2, Quote 7). Some felt that the current culture in maternity services after a baby dies is to ‘deny and defend’ and explained that all they wanted was to ‘feel safe to sit down and just say the truth’ with the staff involved.

#### 3.2.3 Importance of communicating with parents with regular updates

All the parents agreed that communication with regular updates from professionals was extremely important throughout the review process (Table 2, Quote 8). However, they did feel this should be optional and adapted to each parent's needs, as some parents might prefer not to have regular contact (Table 2, Quote 9). A number of parents described how they had an open two‐way channel of communication with staff in the hospital and could ask questions any time, an aspect they said was particularly valuable to them (Table 2, Quote 10). A few parents stated that they had difficulties in establishing contact with professionals, describing how they had to take the initiative, ‘pursue’ contact and meetings. They had to insist on being heard ‘at every turn’ (Table 2, Quote 11). One mother raised a concern about the parents that may not be adept at navigating these communication challenges: ‘what about the parent that isn't willing to do that? Or doesn't even know that that's an option for them?’ (*Interview 2*).

#### 3.2.4 Support for parents

The participants also talked about their experiences of support throughout their bereavement journey. Some felt supported throughout this difficult time and through the hospital review process. Others mentioned how this support was lacking and how they felt they ‘were forgotten’ and felt like ‘just another number’. Most mentioned the concept and the need for someone ‘checking in’ with them, especially after leaving the hospital with the abrupt change from 24 h care to ‘nothing’ and feeling ‘very alone’ (Table 2, Quote 12). Those that had a bereavement midwife ‘check in’ really appreciated this support. A few parents mentioned how this contact should be cautious, and not be insistent (‘…She rang all right … But after that, then she'd text. So, like, if you didn't feel like talking, that was fine. No, she wasn't em, she wasn't pushy at all, at all…’, Table 2, Quotes 13 and 14). A number of parents experienced and valued this support to include guidance through follow‐up meetings and the review process (‘…But in hindsight, only for our bereavement midwife at the time. She guided us through all of that…’, Table 2, Quotes 15 and 16).

### 3.3 On leaving the hospital ‘you're just given so much information inside in the hospital’

#### 3.3.1 Information given to parents (verbal and written)

The first stage of parental involvement in the maternity hospital perinatal death review process began on leaving (or just before) the hospital. The parents spoke about the hospital stay after the birth being about spending time with their baby, making precious memories and their own/their partner's physical recovery. It was on leaving the hospital that they felt the information given to parents regarding what to expect next was ‘so much’ and ‘there's a lot to process already’, for example, the funeral arrangements. A few parents explained that being told a review would take place was welcomed, however, they did not understand at the time what this would entail. Some parents described that providing written material about future meetings and review processes could help avoid this information overload. In addition, they recommended that making a follow‐up call to ensure parents had received all relevant information accurately would be useful (Table 3, Quote 1).

The parents were very clear about what type of information was important for bereaved parents to receive as they were leaving the hospital. They felt it would be important for parents to know what happens after they leave the hospital, who will be in touch and what supports are available to them. Further, the participants thought it would be beneficial to receive clear information and timelines on follow‐up visits, investigations and reviews, and the options they have regarding these (‘I think it should be arranged before you leave the hospital, just to say that this is coming down the line. The results will come back…’, Table 3, Quotes 2 and 3). Seven parents suggested that, ideally, information should be provided in writing in the form of a booklet or information pack (Table 3, Quote 4). They said this would allow bereaved parents to process the information in their own time and ‘soak things in better’ during the initial period after leaving the hospital. Furthermore, the parents wanted to know what local and national support services (i.e., bereavement support, counselling, voluntary support organizations, patient advocacy) are available to them and not to have ‘go looking’ for these supports (‘…have their doctor sit down with them the day they're being discharged and say, look, these are the, you know, supports available to you. And here are the numbers…’, Table 3, Quotes 5 and 6).

#### 3.3.2 Having a point of contact/key contact

Having a point of contact in the hospital (and/or a liaison for the review process) was important to the parents, to have ‘a go‐to person, just that one link person, just one name’ (Table 3, Quotes 7 and 8). They recommended for this contact keep parents updated on investigations/reviews, be available to answer questions and liaise with other professionals (Table 3, Quote 9). The local bereavement midwife fulfilled this role for many of the parents, but this was not always made clear to them when they were leaving the hospital (‘…it probably just needs to be a little clearer to the parents though, like em, who is my contact person if I want to follow up on anything that's happened?…’, Table 3, Quote 10). Some parents thought it was valuable to meet their key contact in person before leaving the hospital so they would ‘have a face to the person’ and that they would have a familiar person who would establish contact with them (‘that it's not some random person that rings you after’, *Interview 7*). One mother indicated that she believed that the workload was too arduous for a single individual: ‘I'm not saying questions weren't answered or people weren't contacted. They were (pause), but they're counselling, they're liaising, they're contacting. That's too much for one person’ (*Interview 1*).

### 3.4 Interaction with hospital ‘waiting in limbo’

#### 3.4.1 The follow‐up meeting for parents

Once the parents had left the hospital and their baby's funeral had taken place, they said they were at home ‘wanting to know what happened to your child’. At this stage many felt the follow‐up meeting for parents with the consultant would be essential to get some answers (‘waiting in limbo for weeks and weeks and not knowing is terrible’, *Interview 16*) and to dispel some misperceptions (‘I had this tightness in my chest all along because you would feel blame, but after that meeting, I felt a lot better’, *Interview 10*) (Table 4, Quote 1). The timing for this follow‐up meeting, according to the parents, needed to be flexible and suit each individual couple, though somewhere between 6 and 12 weeks after the birth was recommended (‘…So I think a time frame of maybe 6 to 12 weeks, or definitely 12 weeks post, was a good time for us…’, Table 4, Quotes 2 and 3). The parents were divided in their opinions regarding the location for the follow‐up meeting, some thought it should be away from the maternity hospital, and others felt going back into the maternity hospital was an opportunity to meet the staff that had cared for them and their baby (‘…the day we went in, we met a nurse that looked after me, we met another midwife. And it really grounded us again, to say that [name of daughter] was real…’, Table 4, Quotes 4 and 5). Ultimately, the consensus was that it should be the parents' choice where they want to attend for their follow‐up visit.

The conduct of this visit and the manner of the consultant can have a huge impact on the parents, either positively (‘it was very reassuring’, *Interview 4*) or negatively (‘that meeting with that man did my mental health no good’, *Interview 12*). Those that had a positive experience were grateful, especially when the meeting had been conducted according to their preferences (Table 4, Quote 6). In contrast, one mother described this visit as an ‘opportunity missed from the hospital to keep the relationship going’ (*Interview 16*). Options regarding the follow‐up visit that participants felt should be offered to parents included meeting a team of professionals rather than one individual, multiple appointments (‘and leave it up to the individual person then to choose to pursue the appointments or not’, *Interview 4*) and having ‘someone neutral’ present (Table 4, Quote 7).

#### 3.4.2 Parents providing feedback to the hospital

When asking the bereaved parents if they would have liked to provide feedback to the hospital on their own and their baby's care, the majority of the parents said they would have liked to, but very few were given the opportunity to do so (Table 4, Quote 8). Parents felt by giving feedback, both positive and negative, they would be able to give their side of events, highlight gaps and/or excellence in care and ultimately help other parents (‘…to give our side of things or what we were unhappy with, or happy with…’, Table 4, Quotes 9 and 10). Again, the consensus was that the timeframe for providing this feedback should be flexible, but around 6–12 weeks after the birth was deemed appropriate (Table 4, Quotes 11 and 12). Parents thought the invitation to provide feedback to the hospital should be both verbally and in writing, with a follow‐up letter or phone‐call to say ‘if you want to opt‐in, if you want to have a chat, we're more than happy to do that’ (*Interview 1*). Many different ways of giving feedback were mentioned. Some thought writing using a questionnaire/feedback form, via email or a letter was appropriate (Table 4, Quote 13). Others thought verbally, over the phone or face‐to‐face, as part of the follow‐up meeting or a separate meeting, was best (Table 4, Quote 14). Many parents felt different options should be offered, so the bereaved parents themselves may choose how to provide feedback (‘…I think there should be whatever option a parent wants, really. Like I don't think it should be restricted to just, you know, contact this number between these times…’, Table 4, Quote 15).

### 3.5 Review itself ‘a way to get answers’

Eight of the 20 parents were informed of a formal review into their care and their baby's death as it was taking place, one further mother learned of the internal review after it had been completed. The other 11 parents were not aware of any formal review taking place. However, all parents had investigations and/or meetings with professionals to identify the cause of death for their babies and any potential contributory factors. In this section, we discuss all these processes together under the term ‘review’ as for the parents the aim and desired outcomes are the same: ‘to try and piece together what exactly did happen’ and ‘to prevent this happening to anybody else’ (if possible).

#### 3.5.1 Aim of reviews

For the parents, the aim of reviews was to get answers, identify errors, prevent events recurring and possibly give them some closure (Table 5, Quotes 1 and 2). Unfortunately, overall the parents felt that what they experienced in the review process was not consistent with this perception (‘…how it came across to us, was almost they were covering themselves…’, Table 5, Quote 3) but rather that it was done to satisfy a predetermined requirement: ‘I really don't see how what's being done at the moment is in any way useful or meaningful, other than just to say that it's been done’ (*Interview 5*).

#### 3.5.2 Parents' contribution to the review

This was also the case in relation to the parents' contribution to the review process. As one mother put it: ‘I think as a parent the review process will mean very little until a parent's voice is heard a bit louder’ (*Interview 14*). All the parents agreed that their contribution to the review process was ‘relevant’, ‘important’ and ‘has to be treated with credibility’. The parents' reasons for contributing to the review were ‘to get the full picture’, ensure all sides of events are recorded, ‘the chronology’, and ultimately so that lessons can be learned (Table 5, Quotes 4 and 5). Suggestions for involving parents appropriately included an invitation to all (‘input at the start and again before it's finalized, so that you can actually see what's been discussed’, *Interview 5*) and a written statement and/or a meeting (‘…when they have seen that written statement, and then maybe set up a meeting with everybody involved…’, Table 5, Quotes 6 and 7). The parents were clear that it should be up to the parents themselves to decide whether to contribute to the review or not, and that ‘the invite should be there anyway’. And what is offered to the parents is followed through (Table 5, Quote 8). Several participants felt that the current process of involving parents is protracted, one father described how ‘the process was so long’ and the effect this had on them (‘…Because every time we went over it, it was heart‐breaking and it was very hard in the days afterwards we found because it is constantly on your mind…’, Table 5, Quote 9). For the parents it was essential that the information they provided was ‘taken with honesty and listened to’ and not ‘dismissed’ or ‘treated as unreliable, uncredible, hearsay’. Some were asked to provide a written statement, as well as attend a meeting for an interview, at times the experience of the review meeting was described as ‘traumatic’, it was particularly distressing if in the end, they realized their input ‘had no impact, it meant nothing’.

#### 3.5.3 Delivering information to parents

The manner of delivering information to parents needs to be clear and compassionate. The parents requested clarity from the beginning regarding when and how results and review reports would be delivered to them. Getting results/reports without prior notice (‘out of the blue’, *Interview 16*) at home was described as ‘disrespectful’ (‘…There was no warning you will get it next week, nothing, there you go, in your in‐box. So to say the least that was extremely hurtful…’, Table 5, Quotes 10 and 11). Many parents preferred receiving reports in person, with professionals facilitating the time and space to process the findings in their own time and ask questions, while also acknowledging their child as a person and their grief (‘…how we're met as parents, grieving parents and that our child is acknowledged throughout the meeting…’, Table 5, Quotes 12 and 13). Some parents stated that bereaved parents need to be offered input to a preliminary review report, rather than being presented with the final version (Table 5, Quote 14). Again, the length of the review process until receiving results/reports were described as ‘long’ and ‘very slow’ by several parents (‘having that hanging over you for months is torturous’, *Interview 7*), especially if there was uncertainty regarding the date of completion (Table 5, Quote 15). A few of the parents did not receive answers to their questions through the review process, and in some instances, freedom of information requests was experienced as necessary to receive missing information (Table 5, Quotes 16 and 17).

#### 3.5.4 Inconsistencies for parents with reviews

The bereaved parents illustrated many inconsistencies with reviews. One mother felt that most parents are not made aware of reviews being started (‘most people don't know that there's a hospital review happen[ing]’, *Interview 5*), and two sets of parents were actually discouraged from pursuing a review (Table 5, Quote 18). Some of the parents explained how they were not appropriately involved in the review process (‘they have to ask you these questions but they don't really want to know the answers’, *Interview 17*) and what they said was not included (Table 5, Quote 19). It required significant effort from the parents to have to persistently contact the review team to ‘be heard’ and for updates (‘it takes so much strength and it takes so much energy’, *Interview 16*). One mother described how she felt ‘shoved off, shoved off’ when asking for updates. Another impact of the review process on parents was a ‘burden of responsibility’ and pressure to ensure recommended changes were implemented in the hospital (‘we felt under enormous pressure to make sure that [em] the proper processes were put in place in the hospital to make sure that wasn't gonna happen *(sic)* to anybody else’, *Interview 11*). The dread of the same issues with care potentially recurring has led to parents feeling a sense of responsibility to ensure recommendations were progressed, solutions found and changes implemented. This added a significant level of pressure and stress to the anxiety they were already experiencing. They felt this was not fair on them and should not have been their responsibility (‘we shouldn't have to do that, we have been through enough, that is not our job’, *Interview 16*).

#### 3.5.5 Outcomes of the reviews for the hospital and the parents

Outcomes of the reviews for the hospital and the parents differed but ultimately the one aim for both families and hospitals was to try and prevent further deaths if possible. The outcomes of the reviews for the hospital, the parents specified, should be learning and change (‘…They are human, they make mistakes but the biggest thing from mistakes is learning from them…’, Table 5, Quote 20). However, several parents experienced their review to be a ‘tick‐box exercise’ and felt that recommendations from the reviews were not implemented (Table 5, Quote 21). The parents said the review process has the potential to get answers, see positive change and have the acknowledgement that their baby's life mattered, and therefore it can help to bring healing and closure (‘…I felt like that was because of our little boy, that he inspired change and that would have been a really lovely thing to hear…’, Table 5, Quotes 22 and 23). One mother summarized the potential outcomes of the review process aptly: ‘it brings comfort and healing at the end of the day when it is done right, and when they are not done right you just have repetitive hurt’ (*Interview 15*).

## 4 DISCUSSION

In Ireland, bereaved parents have been infrequently included in local maternity hospitals' perinatal death review processes.^15^ In this study, we learned from and with bereaved parents, how they may be appropriately involved in these reviews to aid the review process and have their views heard. From the interviews, it was apparent that meaningful parental involvement needs to be considered as a process and not a once‐off meeting where report findings are divulged. Throughout this process, open and clear communication between professionals and the parents is paramount, including unambiguous verbal and written information, as expressed by parents in this study and in line with previous literature.^2^, ^8^ Similarly to what has been reported in previous research, the parents in this study stated that it is essential to have a person as a key contact and support so that they know who to contact with concerns and/or questions once they have left the hospital.^8^ The bereaved parents stated that they want to give feedback to the hospital and the review process, both positive and negative, as well as receive results and reports in a supportive timely manner. The overarching expression from parents in our study was that parental inclusion in reviews needs to be flexible with realistic options available that are sensitive to parents' needs and state of mind, acknowledging their child, their role as his or her parents and their grief. This reflects the findings from earlier studies in this field.^2^

When bereaved parents are meaningfully included in maternity hospitals' perinatal death reviews, they feel their concerns are heard and their views are appreciated.^8^ As expressed in our study and in line with previous research, for parents it is important to understand the circumstances and cause of death of their baby to help to process their grief and potentially plan for future pregnancies.^5^, ^8^ Further, parents in the current study explained how they can provide valuable clinical and nonclinical information to the review process, as well as highlight good or deficient aspects of maternity and/or neonatal care. Previous literature has highlighted the importance of parental contribution to reviews and the value of such participation.^2^, ^8^, ^10^ Collaboration between staff and bereaved parents can result in learning from events and improve services for other parents as indicated, and potentially prevent other perinatal deaths in the future.^10^

Our findings in the Irish setting mirrored many of the findings of the PARENTS 1 and PARENTS 2 studies in the United Kingdom, adding to the growing evidence of the benefits of involving bereaved families in hospitals' mortality reviews.^2^, ^7^, ^8^, ^9^, ^10^, ^24^ Like the participants in the PARENTS 2 study, our cohort of parents emphasized the importance of having a point of contact and the need for personal interaction rather than limited written correspondence.^8^ Further, the concept of someone ‘checking‐in’ was felt to be important by the participants in this study. Not having a designated person in the hospital for parents to contact with questions is an ongoing problem in some Irish maternity units.^25^

In Ireland, parents should be informed if a review into their care and their baby's death is taking place and if not, the reason for not reviewing a death. This has been stipulated in the 2018 national Incident Management Framework.^17^ However, in this study parents were not consistently informed about these reviews. Further, the Incident Framework states that questions from affected persons should be considered as part of the review.^17^ Despite this, many of the parents felt their queries or opinions were not considered appropriately. In the PARENTS 2 study, there were mixed reactions to the feedback form developed for parents to complete after the death of their baby.^8^ Similarly in our study, the parents felt there should be different options available to provide feedback to the hospital in writing or in person, depending on their preferences.

The UK‐PMRT was developed and put into practice to improve the quality of local reviews by incorporating the parents' perspectives and standardizing the review process.^13^ Further research to examine the potential of implementing an electronic review tool like the UK's PMRT in Ireland is warranted, along with identifying barriers and facilitators to its use and uptake in practice. The PARENTS studies highlighted potential challenges to the engagement of bereaved parents in the reviews including the need for endorsement by local management, as well as the provision of extra human resources and support.^10^ Further, it may be difficult to balance the timing of the parental engagement to be sensitive to the parents' grief and fit with the hospital's schedule.^8^ Many of the parents in this study were upset by the protracted nature of reviews in Ireland currently, with it taking many months or even years to receive postmortem and/or review reports. A previous study by the authors showed that 4 of the 19 Irish maternity units released review reports more than 6 months after the event occurred.^18^ Furthermore, recent research examining the timelines in the investigations of 122 stillbirths in Ireland reported the median time from stillbirth to the follow‐up meeting with the consultant with the final report was 140 days (ranging from 54 to 579 days).^26^ The current system with long delays and/or difficulties for parents to get answers and resolution, does not appear to put bereaved parents at the centre of the review process and may be contributing to delayed or complicated grief reactions of parents and families.^16^, ^27^

Since the publication of the National Standards for Bereavement Care Following Pregnancy Loss and Perinatal Death in 2016, the emphasis has been on providing sensitive and individualized bereavement care in Irish maternity hospitals.^28^ Promoting a culture of compassion and honesty is key to this.^28^, ^29^ This culture of openness and compassionate bereavement care needs to continue throughout the review process and is not limited to the bereaved parents' stay in the hospital. It is not acceptable to be unaware and insensitive to parents' emotional and bereavement needs, as every interaction with a member of the hospital staff has the potential to have lasting positive or negative effects.^4^, ^16^, ^27^, ^30^ Regular multidisciplinary, interactive education on communicating and interacting with bereaved parents for all hospital staff would help to foster a compassionate culture.^30^ A workshop (called TEARDROP [Teaching, Excellent, pArent, peRinatal, Deaths‐related, inteRactions, tO, Professionals]) has been developed and evaluated in Ireland, and the aim is to expand this training programme nationally.^30^ TEARDROP consists of six interactive, multidisciplinary workstations covering areas of the National Bereavement Standards to equip staff with skills to provide optimal bereavement care for parents.^28^, ^30^

### 4.1 Practice and policy implications

The current study provides relevant insight and information which can have relevance to practice and the care provided to bereaved parents. Numerous important pieces of information (e.g., contact details, bereavement supports, information on the coronial system) are currently provided to bereaved parents on discharge from the maternity hospital after the birth/death of their baby. Seven of the parents suggested the development of an information booklet explaining the different aspects of the review process (i.e., key contact, supports available, ways to provide feedback, timelines and possible outcomes including results and reports) to complement existing information given to parents. This would be a simple intervention with a potentially large impact on parents. Further, this information booklet should clearly outline the voluntary support organizations and services available to parents.

Realistic timelines for follow‐up meetings and review processes, as well as options for receiving information, results and reports, should be clearly stated to parents and adhered to. Standardization of the local perinatal death review processes at the national level (based on the existing Incident Management Framework), including ways of incorporating parents' views and questions, would be helpful and alleviate discrepancies occurring in reviews and experienced by parents. The development of a review tool like the UK‐PMRT and adaptation of the available associated support material^14^ for the Irish setting may unify review practices further. Regular training sessions for all staff would form part of these standardized practices in Irish maternity hospitals.

A regular system of feedback is important to ensure the meaningful involvement of bereaved parents in review processes is taking place, practices that are sensitive to parents' needs and identify areas in need of further development. Parent experience surveys or a regular audit of parental involvement in maternity hospital perinatal death reviews could provide this feedback.

The findings and implications from this study are transferable to other countries and other mortality reviews, especially child death reviews. Factors affecting the transferability of findings include the availability of specially trained staff, for example, bereavement specialists and resources, that is, time and facilities.

### 4.2 Strength and limitations

Purposeful sampling was implemented to invite bereaved parents in this study with some potential selection bias as participants who were willing to participate in this research likely being those already engaged with maternity services and/or who had raised a previous concern about the review process to parent representatives working within Voluntary Organizations. Efforts were made to include a representative sample across Ireland and invaluable and relevant views on this matter were provided. Due to the need to maintain the anonymity of participants, it was not possible to analyse the findings taking into consideration or adjusting for sociodemographic or obstetric history. Further research with a wider (and perhaps international) sample would be valuable to understand how different individual and family characteristics can impact bereaved parents' experiences.

Both individuals and couples were interviewed. Similar to other research studies,^31^ we found it challenging to recruit fathers to this study with only four taking part, even though the invitation to participate was extended to all partners of those that agreed to be interviewed. Limiting participation to parents who spoke fluent English may have excluded parents from some ethnic minority groups with broader cultural preferences. Due to the COVID‐19 pandemic and public health guidance, all interviews were carried out virtually, which facilitated the geographical widespread representation of participants but hindered the personal rapport between interviewer and interviewee. Further, it excluded participants not comfortable with carrying out an interview virtually.

## 5 CONCLUSIONS

In this study, parents clearly voiced their concerns with and desire to be included in perinatal mortality reviews. A respectful, compassionate and flexible system, tailored to the needs of parents is essential, however, this is not yet consistently present for all bereaved parents in Ireland during their baby's review process. The involvement of parents in reviews needs to be carefully considered and resourced, as poorly managed engagement has the potential to cause more hurt. Causing upset and emotional harm through disrespectful or dismissive comments or practices at any stage of the review process must discontinue. Hearing parents' voices in open transparent collaboration with the hospital staff respects their baby and their grief. It has the potential to both support their healing process and make real differences for parents and babies in the future.

## CONFLICT OF INTEREST

The authors declare that there is no conflict of interest.

### 1 ETHICS STATEMENT

Ethical approval was received from the Clinical Research Ethics Committee of the Cork Teaching Hospitals, Ref. No. ECM 4 (d) 08/09/2020.

## Data Availability

Data are available on request from the authors.
